# Nosocomial bacterial infections and their antimicrobial susceptibility patterns among patients in Ugandan intensive care units: a cross sectional study

**DOI:** 10.1186/s13104-017-2695-5

**Published:** 2017-07-28

**Authors:** Peter Agaba, Janat Tumukunde, J. V. B. Tindimwebwa, Arthur Kwizera

**Affiliations:** Department of Anaesthesia, College of Health Sciences, Makerere University, P. O. Box 7072, Kampala, Uganda

**Keywords:** Drug resistance, Intensive care unit, Nosocomial infections, Mechanical ventilation, Traumatic brain injury

## Abstract

**Background:**

The intensive care unit (ICU) admits critically ill patients requiring advanced airway, respiratory, cardiac and renal support. Despite the highly-specialized interventions, the mortality and morbidity is still high due to a number of reasons including nosocomial infections, which are the most likely complications in hospitalized patients with the rates being highest among ICU patients.

**Methods:**

In this cross-sectional study of 111 adult patients admitted to 2 of the ICUs in Uganda, we set out to describe the commonest bacterial infections, their antimicrobial susceptibility patterns and factors associated with development of a nosocomial infection.

**Results:**

*Klebsiella pneumoniae* (30%), *Acinetobacter species* (22%) and *Staphylococcus aureus* (14%) were the most frequently isolated bacteria. The prevalence of multidrug resistant bacterial species was 58%; 50% *Escherichia coli* and 33.3% *Klebsiella pneumoniae* were extended spectrum beta lactamase or AmpC beta lactamase producers and 9.1% *Acinetobacter species* were extensive drug resistant. Imipenem was the antibiotic with the highest susceptibility rates across most bacterial species. Institution of ventilator support (P 0.003) and severe traumatic brain injury (P 0.035) were highly associated with the development of nosocomial infections.

**Conclusion:**

Due to the high prevalence of multi drug resistant (MDR) and extensive drug resistant bacterial species, there is a need for development of strong policies on antibiotic stewardship, antimicrobial surveillance and infection control to help guide empirical antibiotic therapy and prevent the spread of MDR bacteria and antibiotic drug resistance.

**Electronic supplementary material:**

The online version of this article (doi:10.1186/s13104-017-2695-5) contains supplementary material, which is available to authorized users.

## Background

Nosocomial infections (NIs) are defined as hospital acquired infection developing at least 48–72 h after admission [1]. They are the commonest complications affecting hospitalized patients but are more frequent in intensive care units [2] where outbreaks often originate [3].

Three types of infection account for more than 60% of all nosocomial infections: pneumonia (usually ventilator-associated), urinary tract infection (usually catheter-associated) and primary bloodstream infection (usually associated with the use of an intravascular device) [4]. Antibiotic resistant Gram-positive or negative bacteria including *Staphylococci*, a wide variety of *Enterobacteriaceae*, *Pseudomonas species*, *Acinetobacter species*. account for up to 70% of the nosocomial infections in the ICU patients [5–8].

Five to ten percent of patients admitted to acute care hospitals acquire one or more infections, and the risks have steadily increased during recent decades [9, 10]. Intensive care units represent only 5–15% of hospital beds and account for 10–25% of healthcare costs, corresponding to 1–2% of the gross national product of the United States [6]. A World Health Organization (WHO) systematic review and meta-analysis showed health-care-associated infection density in adult intensive-care units in developing countries was 47.9 per 1000 patient-days (95% CI 36.7–59.1), at least three times as high as densities reported from the USA. In Canada, Zhanel et al., between 1 September 2005 and 30 June 2006, collected 4180 isolates recovered from clinical specimens from patients in 19 intensive care units and found *Staphylococcus aureus* (methicillin sensitive *S. aureus* and methicillin resistant *S. aureus, MRSA*), *Escherichia coli*, *Pseudomonas aeruginosa*, *Haemophilus influenzae*, *Enterococcus species*, *Streptococcus pneumoniae*, and *Klebsiella pneumoniae* were the most common isolates [11].

In a study in San Paulo by Carlos Toufen et al. Population sample of 126 patients found the most frequently isolated bacteria to be *Enterobacteriaceae* (33.8%), *P. aeruginosa* (26.4%), and *S. aureus* (16.9%).

In Kenya, at Kenyatta National Hospital intensive care unit, the most frequently isolated organisms included *P. aeruginosa, Klebsiella, Citrobacter, S. aureus, Staphylococcus pneumoniae, Acinetobacter* and *E. coli* isolated from tracheal aspirate, urine, blood and pus swabs. However, a study done in Mulago Hospital on the prevalence of MRSA among isolates from surgical site infections on the general ward and found a prevalence of 31.5% [12]. This is comparable with 26.9–29.6% prevalence reported in USA, Middle East and other selected African hospitals [13, 14].

For the development of a NI, two pathophysiologic factors must be present: impaired host defences and colonization by pathogenic or non-pathogenic bacteria [1].

Most nosocomial infections arise from the endogenous bacterial flora although many critically ill patients eventually become colonized with resistant bacterial strains. The urinary tract accounts for up to 35–40% of nosocomial infections, which are usually due to Gram-negative organisms and are associated with the use of indwelling catheters or urinary obstruction. Wound infections are the second most common cause, accounting for up to 25–30%. Intravascular catheter-related infections are responsible for 5–10% of intensive care unit infections [15].

Nosocomial pneumonias—the leading cause of death in many intensive care units and the second most common NI, account for another 20–25% and are often caused by Gram-negative organisms [16]. More than 90% of pneumonias are acquired while patients are mechanically ventilated [17]. Mechanical ventilation frequently requires tracheal intubation which, allows aspiration of oral and gastrointestinal material and bacteria [18].

Gastro intestinal bacterial overgrowth with translocation into the portal circulation and retrograde colonization of the upper airway from the gastro intestinal tract followed by aspiration are possible mechanisms for entry for these bacteria [19].

Infection is a leading cause of death in the intensive care unit with mortality rates as high as 60% and twice as much in those patients with a nosocomial infection [20].

The impact of NI on morbidity and mortality is substantial not to mention the effect on increased hospital stay and cost of health care. The increased hospital stay and the need for stronger more expensive drugs mean increased costs for both the patient and the government. This is even more important in resource-limited settings [21, 22]. Medical legal issues may also arise as patients or relatives may blame the ICU staff or the hospital for causing the infection and demand compensation [23].

The global escalation in both community- and hospital-acquired antimicrobial-resistant bacteria is threatening the ability to effectively treat patients [24]. Treatment options are severely limited because these bacteria frequently display multi drug resistance [16]. It is, therefore, conceivable that patients with serious infections will soon no longer be treatable with currently available antimicrobials.

However, this can be prevented. One study showed that one third of nosocomial infections could be prevented through infection control and watchful programs [25].

Practices as simple as hand hygiene have been shown to be quite effective in reducing the rates of NI [26, 27].

Inappropriate empirical antimicrobial therapy is known to adversely affect outcome in severe bacterial infection [28]. Therefore, it is very important for every institution to have local, current microbiological data in order to assess the likely infecting pathogens and the susceptibility patterns. This will facilitate appropriate empirical antimicrobial therapy.

Strict management of antibiotic policies and surveillance programs for resistant organisms, together with infection control procedures, need to be implemented in the ICU and continuously audited.

Although antimicrobial susceptibility patterns are not well documented in Uganda, Anguzu et al in 2007 carried out a study on surgical wound infection that demonstrated resistance to the cheaper more common antibiotics [29]. Therefore, there is a need to develop a national surveillance of antimicrobial resistance patterns.

This study described the common bacterial pathogens and antimicrobial susceptibility pattern among ICU patients in Mulago hospital and International Hospital Kampala.

## Methods

### Study design and setting

This study was a cross sectional study carried out in the intensive care units of Mulago National referral hospital and International Hospital Kampala.

These units have on average a day and night 1:1.5 nurse patient ratio, a critical care specialist and a resident during the day and an anaesthesia resident at night.

### Study population

All patients newly admitted to intensive care units of Mulago hospital and International Hospital Kampala.

### Inclusion criteria

Admission to the intensive care unit.

### Exclusion criteria

Age less than 18 years and patient’s request. If the patient was likely to spend less than 48 h in the ICU.

### Sampling method

The non-probability consecutive sampling method was used on patients newly admitted to the intensive care unit. Patients were selected as they were admitted to the intensive care unit based on the inclusion criteria.

### Consent to participation

A waiver of consent was obtained from the institutional review board since the study posed minimal risk to the patient. In addition, because majority of patients at admission are incapacitated and unable to provide informed consent it would require locating the next of kin which would introduce delays in collecting the base line sample.

### Screening and enrolment

Using the inclusion and exclusion criteria at admission, patients who were eligible had a study number given and their baseline data such as demographics, reason for admission, referring unit recorded and samples such as blood/tracheal aspirate/urine for culture and sensitivity collected.

### Follow-up

After 48–72 h in the intensive care unit, clinically significant samples were taken off for culture and sensitivity. All samples were analysed at a microbiology laboratory.

### Sample collection, handling and processing

Sites selected for blood sampling were swabbed with 70% alcohol. Five to ten millilitres were collected in bactec bottles, transported to the laboratory and placed in the bactec 9120 instruments. Positive bottles were Gram stained and sub cultured and tested for sensitivity by the microbiologist.

Endo-tracheal aspirates were obtained by suctioning the endo tracheal tube or tracheostomy tubes using a sterile suction catheter and the tip cut off with a sterile surgical blade, placed in sterile container, and sent to the laboratory. The most purulent part of the aspirate was used to inoculate plates of blood, chocolate and MacConkey agar by the laboratory technician. Chocolate and blood plates were incubated in carbon dioxide at 35–37 °C and MacConkey in ambient air for 24 h. Positive cultures that had isolates were identified and sensitivity cultures done.

Mid-stream urine or from a sampling port on an indwelling catheter using an aseptic technique was collected in a sterile container. The samples were used to inoculate blood agar and MacConkey agar, which was then incubated at 35–37 °C for 18–24 h. Positive cultures were Gram stained and sub cultured and tested for sensitivities.

Pus or wound swabs from ulcers and wounds that were septic were taken off. In the laboratory blood, MacConkey and chocolate agar were inoculated, incubated, and treated as mentioned above.

As recommended by Clinical and Laboratory Standards Institute (CLSI), isolates were screened for extended spectrum beta lactamase ESBL production using the double disc method and MRSA was identified by the use of cefoxitin disc (30 μg). Multidrug resistance was defined as an isolate non-susceptible to one or more agents in three antimicrobial classes. Extensive drug resistance was defined as non-susceptible to one or more agents in all but two or less antimicrobial classes [30].

Bactec bottles, catalogue number 442192, were purchased from Becton Dickson and company Maryland USA. Antibiotic discs and agar were purchased from Biolab diagnostic laboratory Zrt Budapest Hungary. The catalogue numbers can be found in the Additional file 1.

### Primary study variables

1. The five commonest microbes causing nosocomial infections.
2. Sensitivities of these microbes to the commonly used antibiotics in the intensive care unit such as ceftriaxone, imipenem, piperacillin and tazobactam, gentamicin, ampicillin.

### Secondary study variable

1. Characteristics associated with antimicrobial susceptibility patterns such as, age, sex, and length of antibiotic treatment.

### Sample size

Using the formula N = (Z^2^ u)/e^2^ to estimate risk with a specified precision, a 95% confidence interval, and an estimated risk of acquiring infection of 0.07^9^ and a standard of error of 0.05, sample size calculated was 118.

### Data management and data analysis

Once ethical approval was obtained the questionnaire was tested on 5 patients and corrections were made after discussions with the research assistants, laboratory technician and the statistician. The pre-tested questionnaires were then used to collect both clinical and laboratory data. The principle investigator and research assistants administered questionnaires while laboratory data was transcribed to the questionnaires from laboratory result forms. Data was checked for completeness and accuracy at the end of every day of data collection.

Data was double entered into Epidata version 3.1 with range, consistency and validity checks embedded to ensure accuracy of data. The data was stored on computer hard drive that is password protected to ensure confidentiality and backed up on separate external hard drives kept in separate locations.

Stata version 12 was used for data analysis. This was a descriptive cohort study therefore; the main analysis was descriptive in nature.

### Univariate analysis

The participant baseline characteristics like age, sex, referral status and admission diagnosis were categorized and presented as categorical variables. These were presented as frequencies and their respective proportions in tables, graphs and text.

The rate of acquiring the commonest microbes in nosocomial infections among ICU patients after admission was assessed using methods of survival analysis while the antimicrobial susceptibility patterns to the commonly used antibiotics was presented as frequencies and proportions.

### Bivariate analysis

The rate of occurrence of nosocomial infection antimicrobial susceptibility among chosen patient characteristics in the ICU was assessed using Poisson regression methods. A p value <0.05 was taken as statistically significant.

### Quality control

All research assistants were trained prior to the beginning of the study and during the study.

Questionnaires were tested before the start of actual data collection.

Data cleaning and entry was done on a daily basis and the data was periodically evaluated. All questionnaires were safely stored to enable reference in case of data loss.

## Results

**Fig. 1:**
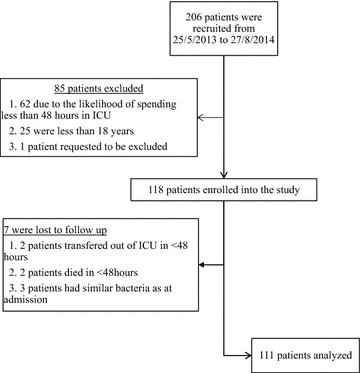
Study profile: two hundred and six patients were recruited. One hundred and eighteen were enrolled and 7 were lost to follow up. Only 111 patients were included in the analysis

Two hundred and six patients were recruited in a period of 15 months and 111 patients were analysed. The difference was due to loss to follow up and exclusion criteria as shown in Fig. 1.

The largest number of patients admitted into the ICU were under the age of 30 years (39.64%), 55.86% were males and 84.68% were referred from within the hospital (Table 1).

**Table 1:** Patient demographics

	N (%)
Age group in years	
≤30	44 (39.64)
31–40	26 (23.42)
41–50	9 (8.11)
51–60	13 (11.71)
>60	19 (17.12)
Sex	
Female	49 (44.14)
Male	62 (55.86)
Referring within hospital	
No	17 (15.31)
Yes	94 (84.68)

**Fig. 2:**
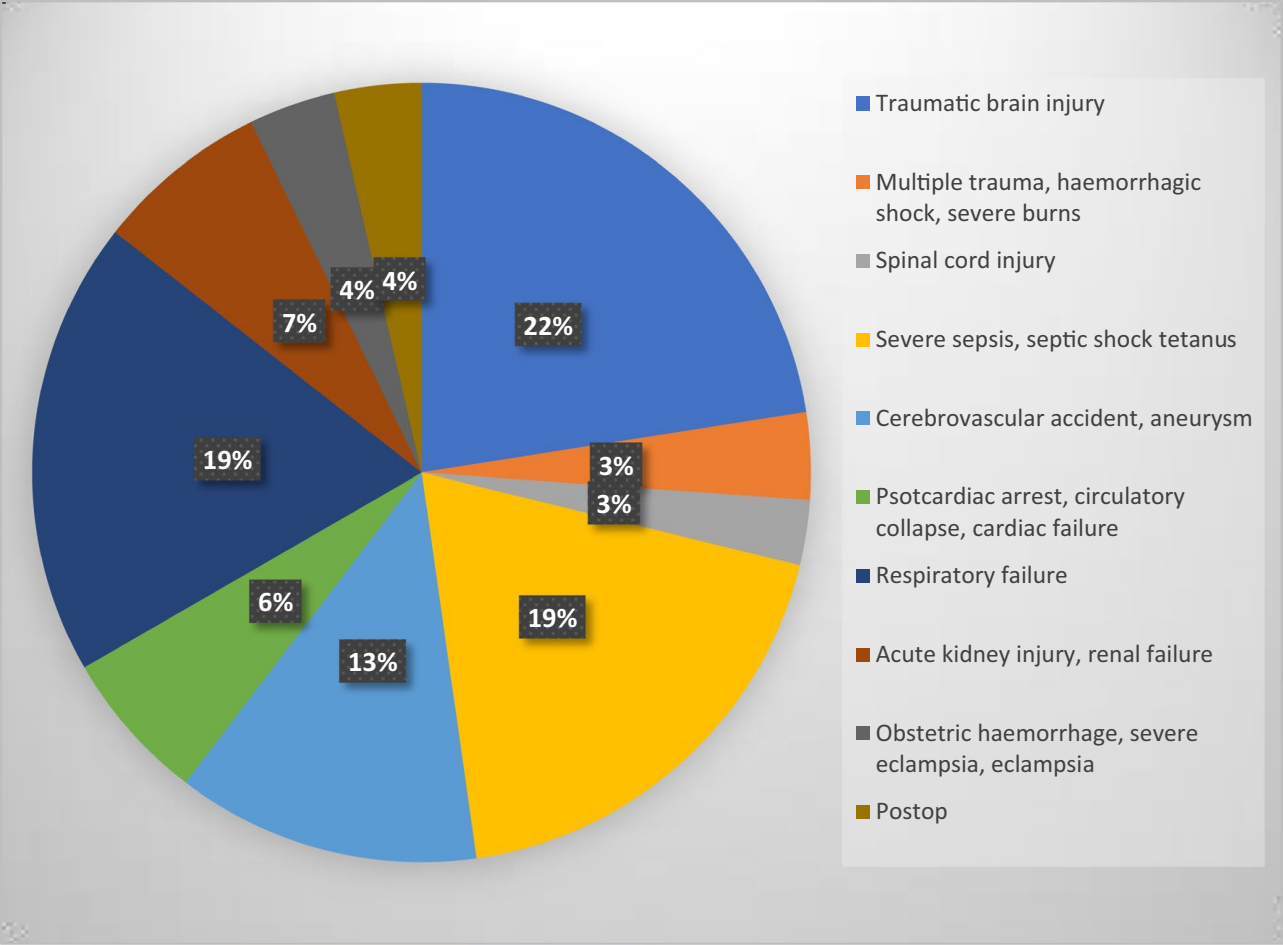
Distribution of admitting diagnosis in percentages. 22% of patients had traumatic brain injury, 19% had a sepsis related diagnosis, 19% respiratory failure and 13% a cerebral vascular accident. The rest fell into the categories of obstetrics, perioperative admissions, spinal cord injury, acute kidney injury or circulatory collapse

Admitting diagnosis included traumatic brain injury (22%), respiratory failure (19%) severe sepsis and septic shock (19%). Others included cerebral vascular accident (CVA), multiple trauma, haemorrhagic shock and acute kidney injury (AKI) (Fig. 2).

At admission, 82 patients were already receiving antibiotics. 72% were receiving cephalosporins alone, 11% were on penicillins alone, 5% on carbapenems alone, 5% on a fluoroquinolone alone, 2% on cephalosporin and metronidazole combined, 2% on macrolides and quinolone, and the rest equally distributed between macrolides, metronidazole and a combination of a carbapenem and metronidazole.

Only one patient had a culture and sensitivity done before giving antibiotics prior to admission into the ICU and no organism was isolated.

Samples taken off for culture and sensitivity at admission were a tracheal aspirate and blood (47% of the patients), blood and urine (2% of the patients), a tracheal aspirate blood and urine (2% of the patients), blood alone (37% of the patients), tracheal aspirate alone in 12% of the patients. There were no wound swabs done.

Organisms isolated at admission into the ICU include *K. pneumoniae* (11.94%) and *E. coli* (7.46%) in the tracheal aspirates, *S*. *aureus* (5%) and coagulase negative *S. aureus* (2.02%) in blood. No organisms were isolated in urine (Table 2). Their antibiotic susceptibility patterns are shown in Table 3.

**Table 2:** Organisms isolated from samples taken off at admission to the ICU

Bacterial species	Sample		
	Blood	Tracheal	Urine
	N = 99	N = 67	N = 4
	N (%)	N (%)	N (%)
*Klebsiella pneumoniae*	1 (1.00)	8 (11.94)	0
*Staphylococcus aureus*	5 (4.40)	3 (4.48)	0
*Acinetobacter* spp.	1 (1.00)	4 (5.97)	0
*Pseudomonas aeruginosa*	0	3 (4.48)	0
*Enterobacter* spp.	0	2 (2.99)	0
*Escherichia coli*	0	5 (7.46)	0
*MRSA*	0	0	0
*Citrobacter freundi*	1 (1.00)	0	0
*Coagulase negative staphylococcus*	2 (2.02)	1 (1.49)	0
*Proteus mirabilis*	0	1 (1.49)	0
*Viridans streptococcus*	0	0	0
*Enterococcus* spp.	0	0	0

**Table 3:** Susceptibility rates for organisms isolated from samples taken off at admission to the ICU

	Bacterial species from blood sample				Bacterial species from tracheal sample							
	No. susceptible/total number tested (%)				No. susceptible/total number tested (%)							
	*K. pneu*	*S. aureus*	*A.* spp.	*C. freundi*	*K. pneu*	*S. aureus*	*E. coli*	*Acineto*	*Pseudo*	*Proteus*	*Enterobacter*	*Coagulase*
Amikacin (30 µg)	1/1 (100)	–	0/1 (0)	1/1 (100)	1/2 (50)	–	–	2/2 (100)	3/3 (100)	–	–	–
Augmentin (20/10 µg)	–	–	–	0/1 (0)	0/6 (0)	–	1/5 (20)	–	–	0/1 (0)	0/2 (0)	–
Ampicillin (10µ)	–	–	–	0/1 (0)	0/7 (0)	–	0/5 (0)	–	–	0/1 (0)	0/2 (0)	–
Cefotaxime (30 µg)	0/1 (0)	–	–	–	0/6 (0)	–	0/1 (0)	–	–	0/1 (0)	1/2 (50)	
Cefuroxime (30 µg)	0/1 (0)	–	–	0/1 (0)	0/6 (0)	0/2 (0)	0/5 (0)	–	–	0/1 (0)	0/2 (0)	–
Ceftazidime (30 µg)	0/1 (0)	–	1/1 (100)	0/1 (0)	1/6 (16.67)	0/1 (0)	0/4 (0)	1/1 (100)	2/2 (100)	0/1 (0)	1/2 (50)	–
Ceftriaxone (30 µg)	0/1 (0)	–	–	0/1 (0)	0/6 (0)	1/1 (100)	0/5 (0)	–	–	–	0/1 (0)	–
Cefepime (30 µg)	–	–	–	–	–	–	–	1/1 (100)	1/1 (100)	–	–	–
Ciprofloxacin (5 µg)	0/1 (0)	1/3 (33.33)	–	–	2/6 (33.33)	0/3 (0)	0/4 (0)	1/1 (100)	2/2 (100)	0/1 (0)	1/2 (50)	0/1 (0)
Chloramphenicol (5 µg)	0/1 (0)	1/3 (33.33)	–	–	2/7 (28.57)	–	0/4 (0)	–	–	0/1 (0)	1/2 (50)	0/1 (0)
Co-trimoxazole (1.25/23.5 µg)	0/1 (0)	0/4 (0)	–	0/1 (0)	0/7 (0)	0/2 (0)	0/5 (0)	0/3 (0)	–	0/1 (0)	0/2 (0)	0/1 (0)
Erythromycin (15 µg)	–	0/4 (0)	–	–	–	0/2 (0)	–	–	–	–	–	1/1 (100)
Oxacillin (1 µg)	–	2/3 (66.66)	–		–	0/2 (0)	–	–	–	–	–	1/1 (100)
Tetracycline (30 µg)	–	1/1 (100)	0/1 (0)	–	–	2/2 (100)	–	–	–	–	–	1/1 (100)
Penicillin G (10 µg)	–	0/2 (0)	–	–	–	–	–	–	–	–	–	0/1 (0)
Gentamicin (10 µg)	0/1 (0)	2/2 (100)	–	0/1 (0)	0/4 (0)	1/1 (100)	1/5 (20)	2/2 (100)	3/3 (100)	0/1 (0)	1/2 (0)	–
Imipenem (10 µg)	1/1 (100)	–	–	1/1 (100)	7/7 (100)	1/1 (100)	5/5 (100)	–	1/1 (100)	1/1 (100)	1/1 (100)	–
Meropenem (10 µg)	–	–	–	–	–	–	–	–	–	–	1/1 (100)	–
Vancomycin (30 µg)	–	1/1 (100)	–	–	–	0/1 (0)	–	–	–	–	–	–

*K. pneu, Klebsiella pneumoniae; S. aureus, Staphylococcus aureus; A.* spp.*, Acinetobacter species; C. freundi, Citrobacter freundi; Acineto, Acinetobacter species; Proteus, Proteus mirabilis; Enterobacter, Enterobacter species; Coagulase, Coagulase negative Staphylococcus*

After 48–72 h of admission into the ICU, 32 patients developed a nosocomial infection and a total of 52 isolates were obtained. 20 of these patients grew one organism, 11 grew two organisms and only one patient grew 3 organisms. Samples taken of for analysis were blood and tracheal aspirates alone in 38 and 12% of the patients respectively, a combination of blood and urine, blood and tracheal aspirate in 1 and 47% of the patients respectively, and 2% of patients had blood, urine and tracheal aspirates taken off for analysis.

Organisms isolated (no of isolates) were *K*. *pneumoniae* (15), *Acinetobacter species* (11), *S. aureus* (7), *P*. *aeruginosa* (6), *Enterobacter species* (5), *E coli* (2), coagulase negative *Staphylococcus* (3), *Streptococcus viridans* (1) and *Enterococcus species* (1). Their distribution in blood and tracheal is further shown in Table 4.

**Table 4:** Bacterial organism isolated from samples taken off after 48–72 h of admission

Bacterial species	No of isolates 52	Sample		
		Blood	Tracheal	Urine
		N = 93	N = 59	N = 3
	N (%)	N (%)	N (%)	N (%)
*Klebsiella pneumoniae*	15 (28.8)	2 (2.15)	13 (22.03)	0
*Staphylococcus aureus*	7 (13.5)	3 (3.23)	4 (6.78)	0
*Acinetobacter species*	11 (21.2)	0	11 (18.64)	0
*Pseudomonas aeruginosa*	6 (11.5)	1 (1.08)	5 (8.47)	0
*Enterobacter species*	5 (9.6)	1 (1.08)	4 (6.78)	0
*Escherichia coli*	2 (3.8)	1 (1.08)	1 (1.69)	0
*MRSA*	1 (1.9)	1 (1.08)	0	0
*Citrobacter freundi*	0	0	0	0
*Coagulase negative staphylococcus*	3 (5.8)	3 (3.23)	0	0
*Proteus mirabilis*		0	0	0
*Viridans streptococcus*	1 (1.9)	1 (1.08)	0	0
*Enterococcus species*	1 (1.9)	1 (1.08)	0	0

**Fig. 3:**
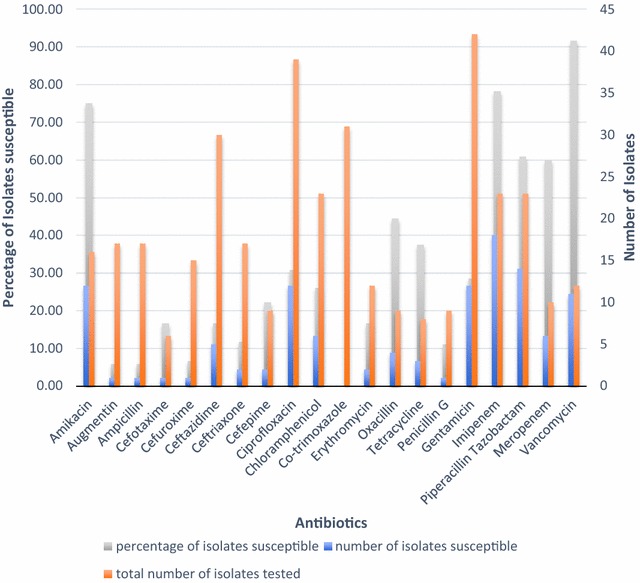
Overall susceptibility patterns of organisms isolated at 48–72 h. The graph shows the proportion of isolates susceptible to each antibiotic and the number of isolates tested against each antibiotic

Antimicrobial susceptibility patterns of the 9 isolated organisms were then analysed according to the samples taken off. Generally, the highest susceptibility (Table 5; Fig. 3) rates were found to be to the amikacin, vancomycin and imipenem and the lowest rates were to cephalosporins, ciprofloxacin and gentamycin. Break down of susceptibility patterns according to sample is shown in Tables 5 and 6.

**Table 5:** Overall susceptibilities of organisms isolated from samples taken off after 48–72 h of admission

	Bacterial species									
	No. susceptible/total number tested (%)									
	*K. pneumoniae*	*S. aureus*	*Pseudomonas* spp.	*Acinetobacter* spp.	*Enterobacter* spp.	*E. coli*	*MRSA*	*Coagulase negative Staph*	*Viridans streptococcus*	*Enterococcus*
Amikacin (30 µg)	3/3 (100)	0/1 (0)	2/5 (40)	4/6 (66.67)	2/3 (67)	1/1 (100)	–	–	–	–
Augmentin (20/10 µg)	1/10 (10)	–	–	0/2 (0)	0/4 (0)	0/1 (0)	–	–	–	–
Ampicillin (10 µg)	0/11 (0)	–	–	0/1 (0)	0/4 (0)	–	–	–	–	1/1 (100)
Cefotaxime (30 µg)	1/4 (25)	–	–	0/2 (0)	–	–	–	–	–	–
Cefuroxime (30 µg)	1/11 (9.10)	–	–	–	0/4 (0)	–	–	–	–	–
Ceftazidime (30 µg)	1/9 (11.11)	0/1 (0)	2/5 (40)	2/10 (20.00)	0/4 (0)	0/1 (0)	–	–	–	–
Ceftriaxone (30 µg)	1/8 (12.50)	1/1 (100)	–	0/3 (0)	0/4 (0)	0/1 (0)	–	–	–	–
Cefepime (30 µg)	–	–	1/4 (25)	1/5 (20)	–	–	–	–	–	–
Ciprofloxacin (5 µg)	3/9 (33.33)	3/6 (50)	3/6 (50)	1/8 (12.05)	1/4 (25)	0/1 (0)	1/1 (100)	0/3 (0)	–	0/1 (0)
Chloramphenicol (30 µg)	2/9 (22.22)	3/4 (75)	–	0/2 (0)	0/4 (0)	–	0/1 (0)	0/1 (0)	1/1 (100)	0/1 (0)
Co-trimoxazole (1.25/23.5 µg)	0/10 (0)	0/4 (0)	–	0/8 (0)	0/5 (0)	0/1 (0)	0/1 (0)	0/2 (0)	–	–
Erythromycin (15 µg)	–	1/6 (16.67)	–	–	0/2 (0)	–	0/1 (0)	0/2 (0)	1/1 (100)	–
Oxacillin (1 µg)	–	1/4 (25)	–	–	2/2 (100)	–	0/1 (0)	1/2 (50)	–	–
Tetracycline (30 µg)	0/1 (0)	2/3 (66.67)	0/1 (0)	0/1 (0)	–	–	–	1/1 (100)	–	0/1 (0)
Penicillin G (10 µg)		1/4 (25)	–	–	0/2 (0)	–	–	0/2 (0)	0/1 (0)	–
Gentamicin (10 µg)	2/10 (20.00)	4/6 (66.67)	3/6 (50)	1/9 (11.11)	0/5 (0)	0/1 (0)	1/1 (100)	0/3 (0)	–	1/1 (100)
Imipenem (10 µg)	5/8 (62.5)	1/1 (100)	4/5 (80)	5/6 (83)	2/2 (100)	1/1 (100)	–	–	–	–
Piperacillin tazobactam (100/10 µg)	4/4 (100)	–	3/6 (50)	5/9 (55.56)	2/4 (50)	–	–	–	–	–
Meropenem (10 µg)	0/2 (0)		1/1 (100)	2/4 (50.00)	3/3 (100)	–	–	–	–	–
Vancomycin (30 µg)	–	5/6 (83.3)	–	–	–	–	1/1 (100)	2/2 (100)	1/1 (100)	1/1 (100)

**Table 6:** Multidrug resistant organism isolated after 48–72 h in the ICU

Organism	No. of MDR isolates/total no. of isolates	% of isolates that were MDR
*Klebsiella pneumoniae*	11/15	73.3
*Acinetobacter species*	7/11	63.6
*P. aeruginosa*	3/6	50.0
*Staphylococcus aureus*	4/7	57.1
*Enterobacter species*	3/5	60.0
*Escherichia coli*	1/2	50.0
Overall	29/50	58.0

Twenty nine of the 52 isolates were MDR organisms. In Table 6, one isolate of *Acinetobacter species* was extensively drug resistant (XDR). Three isolates of *K*. *pneumoniae* and one of *E. coli* was extended spectrum beta lactamase (ESBL) producers while one isolate of *K. pneumoniae* was AmpC beta lactamase (AmpC BL) producer. There was only one isolate of MRSA.

Mechanical ventilation and traumatic brain injuries were highly associated with the risk of developing a nosocomial infection with values of 0.003 and 0.035 respectively. The age bracket 41–50 years had an observed higher risk of infection odds ratio 0.27 (confidence interval 0.03–2.39) and was statistically significant (p value of 0.0412; Table 7).

**Table 7:** Associated factors for infection after 48 h of admission in the ICU

Risk factors	Not infected	Infected	Odds ratio (95% CI)	P value
	N (%)	N (%)		
Age group in years				
≤30	30 (37.97)	14 (43.75)	1.27 (0.55–2.92)	0.723
31–40	18 (22.78)	8 (25.00)	1.13 (0.43–2.94)	0.983
41–50	8 (9.86)	1 (3.13)	0.27 (0.03–2.39)	0.0412
51–60	10 (10.13)	3 (9.38)	0.71 (0.18–2.78)	0.901
>60	13 (16.46)	6 (18.75)	1.17 (0.40–3.41)	0.969
Sex				
Female	34 (43.04)	15 (46.88)		
Male	45 (56.96)	17 (53.13)	0.86 (0.38–1.95)	0.717
Referring within hospital				
No	12 (15.19)	5 (15.63)		
Yes	67 (84.81)	27 (84.38)	0.97 (0.31–3.01)	0.935
Admitting diagnosis				
Traumatic brain injury	13 (16.46)	12 (37.50)	3.05 (1.20–7.72)	*0.035*
Haemorrhage	2 (2.53)	2 (6.25)	2.57 (0.35–19.05)	0.653
Spinal cord injury	2 (2.53)	1 (3.13)	1.242 (0.11–14.20)	0.843
Severe sepsis	18 (22.78)	3 (9.38)	0.35 (0.10–1.29)	0.163
Cerebrovascular accident	9 (11.39)	5 (15.63)	1.44 (0.44–4.69)	0.747
Cardiac condition	7 (8.86)	0	–	–
Respiratory condition	18 (22.78)	3 (9.38)	0.35 (0.10–1.29)	0.163
Kidney condition	5 (6.33)	3 (9.38)	1.53 (0.34–6.82)	0.837
Obstetric condition	1 (1.27)	3 (9.38)	8.07 (0.81–80.71)	0.143
Post Operative	4 (5.06)	0	–	–
Antibiotics before admission				
No	18 (22.78)	4 (12.50)		
Yes	61 (77.22)	28 (87.50)	2.07 (0.64–6.67)	0.334
Ventilation				
No	43 (54.43)	7 (21.88)		
Yes	36 (45.57)	25 (78.13)	4.27 (1.65–11.01)	*0.003*

## Discussion

This is the first study of nosocomial bacterial infections in the ICU to be conducted in Uganda so there is no local data to compare with. Of 118 recruited patients, 111 were analysed and 50 isolates were obtained from 32 patients. Specimens were mainly from the blood stream and the trachea. Previous studies have documented that close to half of isolates in African ICUs are respiratory followed by abdominal and blood stream with urinary infections coming fourth [20]. This study was designed to collect bacterial isolates to study antimicrobial susceptibility patterns thus cannot be used to make the same conclusions.

Although studies have shown a trend towards greater proportion of Gram-positive infection, this study found the majority of isolates were Gram negative which supports findings from the extended prevalence of infection in intensive care (EPIC II) study [20].

This study found that the commonest Gram-negative organisms were *K. pneumoniae*, *Acinetobacter* species and *P. aeruginosa* while *S. aureus* was the commonest Gram-positive organism. This is in agreement with the EPIC II study in African ICUs especially the proportionately greater number of *Acinetobacter species* isolates [20]. *Acinetobacter* is known to be present in water supplies of hospitals [31], and contaminates resuscitation equipment and reusable ventilator circuits [32]. This suggests it can be prevented by simple infection control measures such sterilizing resuscitation equipment, reusable ventilator circuits and avoiding using tap water to flush nasogastric tube [20, 32].

More than a quarter of *K. pneumoniae* isolates were ESBL producers, half of the *E. coli* isolates were AmpC beta lactamase producers and only one isolate was MRSA. This low rate of MRSA is unusual and is probably due to low proportion of samples from wounds and surgical sites as these are the sights commonly infected by MRSA [11].

Of the 52 isolates, only 1 isolate of *Acinetobacter* showed extensive drug resistance (XDR). A study on surgical site infection at Mulago National Hospital showed somewhat higher proportions of MDR and ESBL. However, this was in the obstetrics and gynaecology, general surgery and orthopaedic wards [33]. ICU rates are expected to be higher considering that it’s a confluence of these wards. However, missed ICU opportunities are high and traumatic brain injuries (TBIs) take up a significant proportion of all admissions [34].

The highest susceptibility rates recorded for *K. pneumoniae, P. aeruginosa* and *Acinetobacter* species were to amikacin (with the exception of *P. aeruginosa*) and imipenem while the lowest susceptibility rates were to ampicillin, ceftriaxone, ceftazidime, ciprofloxacin and gentamicin. This is similar to findings in a study on antimicrobial resistance of Gram-negative bacilli among ICU patients in the USA [16]. However, a study in Kenyatta national hospital found much better susceptibility to ceftazidime and this might be explained by a difference in prescribing practice [35]. The susceptibility rates to piperacillin and tazobactam were on average about 50%, less than expected probably because of its increasing use [36]. The beta lactamase producers were susceptible to amikacin and imipenem. The available data show that carbapenems are the most active agents against ESBL *Enterobacteriaceae* however, the data for aminoglycosides is sparse and one review of 85 episodes of bacteraemia showed 71% of isolates were resistant to aminoglycosides [37, 38].

*Staphylococcus aureus* had the highest susceptibility to chloramphenicol, tetracycline and vancomycin while it was non-susceptible to cotrimoxazole, erythromycin, penicillin and oxacillin. The other Gram-positive isolates were all susceptible to vancomycin. MRSA was susceptible to amikacin, gentamicin and vancomycin. These findings are consistent with the study on surgical site wound infections in Mulago [33]. This may suggest that the organisms have a common origin. A study by Seni et al. to characterise the lineages of *S. aureus* among patients with surgical site infection at Mulago found two predominate lineages clonally circulating on the surgical wards and three others confined to the obstetric wards [39]. So, it is plausible that the patterns of susceptibility seen in the ICU maybe do to single clones.

This study found an association between ventilation and development of an NI 48–72 h after admission. A patient was more likely to develop a NI if they were given ventilator support odds ratio 4.43 and was statistically significant (p value of 0.002). This is not surprising as ventilator support was invasive and is similar to findings in a number of studies all over the world [20, 40]. There is a relationship between severe traumatic brain injury and development of a nosocomial infection odds ratio 3.05 and p value 0.03. These patients were usually intubated and on mechanical ventilation. In addition, the age bracket 41–50 years had an observed higher risk of infection odds ratio 0.27 (confidence interval 0.03–2.39) and was statistically significant (p value of 0.0412). The risk of infection is greater with advanced age especially over the age of 60 and this is because of impaired immune system with extremes of age [41, 42]. However, in this study there was no observed increase in risk of infection with patients above 60 years.

## Limitations

We excluded patients under 18 years who would have made a significant contribution to the study population.

The majority of patients, 72%, were on antibiotics prior to admission and its plausible that the prevalence of bacteria seen is not an accurate estimate of the truth. The chances of identifying bacteria are greater when samples are taken off before antibiotics are given. We however, found there was an increased risk of infection in these patients that didn’t reach statistical significance (Table 7).

## Conclusion

The findings suggest that the commonest bacterial NI are *K. pneumoniae, Acinetobacter species, S. aureus, P. aeruginosa, Enterobacter species,* coagulase negative *Staphylococcus, E. coli* and *Enterococcus species*. The prevalence of MDR bacterial species was 58.0% with 50% of *E. coli* and 33.3% of *K. pneumoniae* ESBL or AmpC BL producers and 9.1% of *Acinetobacter species* XDR. Imipenem and amikacin are the antibiotics with the highest susceptibility rates across most bacterial species. Institution of ventilatory support and severe traumatic brain injury are associated with increased risk for the development of NI.

The increased rates of *Acinetobacter* in our ICUs compared to ICUs in Europe and North America are an indication of difference in practice and mean simple change in practice such as sterilization of ambu bags and breathing circuits can reduce the rates of this infection. There is a need for increased funding for healthcare and stricter policies on infection control practices such as hand washing and infection control bundles. The difference in infection rates across ICUs in the country should be investigated and an audit of infection control practices should be carried out.

The prescription of carbapenems should be restricted to initial management of serious bacterial infection in which MDR organism are suspected and should be avoided in situations were a narrow spectrum antibiotic would be equally effective. Antibiograms in the ICU are needed to assess local susceptibility patterns and aid in selecting empiric antibiotic therapy and in monitoring resistance trends in the hospital. There is a need for development of strong policies on antibiotic stewardship, antimicrobial surveillance and infection control to prevent the spread of MDR bacteria and antibiotic drug resistance.

## Additional file

**Additional file 1.** Questionnaire: This is a copy of the questionnaire used throughout the study to collect data.

## Abbreviations

HAI: hospital acquired infection
NI: nosocomial infection
ICU: intensive care unit
USA: United States of America
MRSA: methicillin resistant *Staphylococcus aureus*
MDR: multidrug resistant
XDR: extensively drug resistant
ESBL: extended spectrum beta lactamase
BL: beta lactamase
WHO: World Health Organization
CLSI: Clinical and Laboratory Standards Institute
CVA: cerebral vascular accident
AKI: acute kidney injury
Clinically significant sample: samples from the site or suspected site of infection for example a tracheal aspirate in a case of ventilator associated pneumonia
Nosocomial infection: hospital acquired infection developing at least 48 h after admission
