# Supplementary material for: Nosocomial bacterial infections and their antimicrobial susceptibility patterns among patients in Ugandan intensive care units: a cross sectional study
Source: BMC Res Notes. 2017 Jul 28;10:349. doi: 10.1186/s13104-017-2695-5 (PMC5534037; doi:10.1186/s13104-017-2695-5)
Supplement: Supplementary file 1 — Additional file 1. Questionnaire: This is a copy of the questionnaire used throughout the study to collect data. [file 13104_2017_2695_MOESM1_ESM.docx]

COMMON NOSOCOMIAL BACTERIAL INFECTIONS AND ANTIMICROBIAL SUSCEPTIBILITY PATTERNS AMONG PATIENTS IN THE INTENSIVE CARE UNIT

1. Study site Mulago / IHK
2. Study no.____________ IP.NO. _________ Date______ Sex____ Age_____
3. Referring Unit __________ Referral within the hospital: Yes or No
4. Antibiotics prior to admission: No

Yes

1. Specify the class of antibiotics

a. Cephalosporins b. penicillins c. carbapenem d. macrolides

e. fluoroquinolones

1. Culture & sensitivity prior to admission: Yes

No

1. Was culture sample taken before giving antibiotics: Yes or No
2. What organism was isolated? _________________________________
3. What was the sensitivity profile? ______________________________

| **Antibiotic** | **Sensitive**  **Yes 0r No** |
| --- | --- |
| Imepinenem/Meropenem |  |
| Piperacillin & Tazobactam |  |
| ceftriaxone |  |
| cefotaxime |  |
| cefuroxime |  |
| ceftazidime |  |
| ciprofloxacin |  |
| ampicillin |  |
| gentamicin |  |

1. What is the admitting diagnosis? _________________________
2. Does the patient have
3. Evidence of or suspected site of infection
4. Total white cell count <4 or >12 * 10^9
5. Heart rate >90 bpm
6. Respiratory rate >20brpm
7. Temperature <36 or >38.3 ^0^C
8. Base line sample taken for culture

a. Blood b. tracheal aspirate c. midstream urine d. wound swab

1. Results for baseline culture: Isolate:
   1. Blood 1^st^ ______________________ 2^nd^ _______________________
   2. Tracheal 1^st^____________________ 2^nd^ ______________________
   3. Urine1st_______________________ 2^nd^ ______________________
   4. Wound swab1st____________________ 2^nd^ _____________________

**Sensitivity profile**

| **Blood**  **Antibiotic** | **Isolate sensitivity** | | **Tracheal Antibiotic** | **Isolate sensitivity** | |
| --- | --- | --- | --- | --- | --- |
|  | **1^st^ S or R** | **2^nd^ S or R** |  | **1^st^ S or R** | **2^nd^ S or R** |
| Amikacin |  |  |  |  |  |
| Augmentin |  |  |  |  |  |
| Ampicillin |  |  |  |  |  |
| Cefotaxime |  |  |  |  |  |
| Cefuroxime |  |  |  |  |  |
| Ceftazidime |  |  |  |  |  |
| Ceftriaxone |  |  |  |  |  |
| Cefepime |  |  |  |  |  |
| Ciprofloxacin |  |  |  |  |  |
| Chloramphenicol |  |  |  |  |  |
| Co-trimoxazole |  |  |  |  |  |
| Erythromycin |  |  |  |  |  |
| Oxacillin |  |  |  |  |  |
| Tetracycline |  |  |  |  |  |
| Penicillin G |  |  |  |  |  |
| Gentamicin |  |  |  |  |  |
| Imepenem |  |  |  |  |  |
| Piperacillin Tazobactam |  |  |  |  |  |
| Meropenem |  |  |  |  |  |
| Vancomycin |  |  |  |  |  |

| **Urine**  **Antibiotic** | **Isolate sensitivity** | | **Swab**  **Antibiotic** | **Isolate sensitivity** | |
| --- | --- | --- | --- | --- | --- |
|  | **1^st^ S or R** | **2^nd^ S or R** |  | **1^st^ S or R** | **2^nd^ S or R** |
| Amikacin |  |  | Amikacin |  |  |
| Augmentin |  |  | Augmentin |  |  |
| Ampicillin |  |  | Ampicillin |  |  |
| Cefotaxime |  |  | Cefotaxime |  |  |
| Cefuroxime |  |  | Cefuroxime |  |  |
| Ceftazidime |  |  | Ceftazidime |  |  |
| Ceftriaxone |  |  | Ceftriaxone |  |  |
| Cefepime |  |  | Cefepime |  |  |
| Ciprofloxacin |  |  | Ciprofloxacin |  |  |
| Chloramphenicol |  |  | Chloramphenicol |  |  |
| Co-trimoxazole |  |  | Co-trimoxazole |  |  |
| Erythromycin |  |  | Erythromycin |  |  |
| Oxacillin |  |  | Oxacillin |  |  |
| Tetracycline |  |  | Tetracycline |  |  |
| Penicillin G |  |  | Penicillin G |  |  |
| Gentamicin |  |  | Gentamicin |  |  |
| Imepenem |  |  | Imepenem |  |  |
| Piperacillin Tazobactam |  |  | Piperacillin Tazobactam |  |  |
| Meropenem |  |  | Meropenem |  |  |
| Vancomycin |  |  | Vancomycin |  |  |

1. 48-72 hours later laboratory sample taken for culture and sensitivity
   1. Blood b. tracheal aspirate c. midstream urine d. wound swab
2. Results: Isolate:
   1. Blood1st______________________ 2^nd^ ______________________
   2. Tracheal 1st______________________ 2^nd^ ______________________
   3. Urine1st______________________ 2^nd^ ______________________
   4. Wound swab1st____________________ 2^nd^ _____________________

**Sensitivity profile**

| **Blood**  **Antibiotic** | **Isolate sensitivity** | | **Tracheal**  **Antibiotic** | **Isolate sensitivity** | |
| --- | --- | --- | --- | --- | --- |
|  | **1^st^ S or R** | **2^nd^ S or R** |  | **1^st^ S or R** | **2^nd^ S or R** |
| Amikacin |  |  | Amikacin |  |  |
| Augmentin |  |  | Augmentin |  |  |
| Ampicillin |  |  | Ampicillin |  |  |
| Cefotaxime |  |  | Cefotaxime |  |  |
| Cefuroxime |  |  | Cefuroxime |  |  |
| Ceftazidime |  |  | Ceftazidime |  |  |
| Ceftriaxone |  |  | Ceftriaxone |  |  |
| Cefepime |  |  | Cefepime |  |  |
| Ciprofloxacin |  |  | Ciprofloxacin |  |  |
| Chloramphenicol |  |  | Chloramphenicol |  |  |
| Co-trimoxazole |  |  | Co-trimoxazole |  |  |
| Erythromycin |  |  | Erythromycin |  |  |
| Oxacillin |  |  | Oxacillin |  |  |
| Tetracycline |  |  | Tetracycline |  |  |
| Penicillin G |  |  | Penicillin G |  |  |
| Gentamicin |  |  | Gentamicin |  |  |
| Imepenem |  |  | Imepenem |  |  |
| Piperacillin Tazobactam |  |  | Piperacillin Tazobactam |  |  |
| Meropenem |  |  | Meropenem |  |  |
| Vancomycin |  |  | Vancomycin |  |  |

| **Urine**  **Antibiotic** | **Isolate sensitivity** | | **Swab**  **Antibiotic** | **Isolate sensitivity** | |
| --- | --- | --- | --- | --- | --- |
|  | **1^st^ S or R** | **2^nd^ S or R** |  | **1^st^ S or R** | **2^nd^ S or R** |
| Amikacin |  |  | Amikacin |  |  |
| Augmentin |  |  | Augmentin |  |  |
| Ampicillin |  |  | Ampicillin |  |  |
| Cefotaxime |  |  | Cefotaxime |  |  |
| Cefuroxime |  |  | Cefuroxime |  |  |
| Ceftazidime |  |  | Ceftazidime |  |  |
| Ceftriaxone |  |  | Ceftriaxone |  |  |
| Cefepime |  |  | Cefepime |  |  |
| Ciprofloxacin |  |  | Ciprofloxacin |  |  |
| Chloramphenicol |  |  | Chloramphenicol |  |  |
| Co-trimoxazole |  |  | Co-trimoxazole |  |  |
| Erythromycin |  |  | Erythromycin |  |  |
| Oxacillin |  |  | Oxacillin |  |  |
| Tetracycline |  |  | Tetracycline |  |  |
| Penicillin G |  |  | Penicillin G |  |  |
| Gentamicin |  |  | Gentamicin |  |  |
| Imepenem |  |  | Imepenem |  |  |
| Piperacillin Tazobactam |  |  | Piperacillin Tazobactam |  |  |
| Meropenem |  |  | Meropenem |  |  |
| Vancomycin |  |  | Vancomycin |  |  |

1. At time of taking the blood culture sample

White cell count______

Heart rate_____

Respiratory rate____

Temperature_____

Ph___

PCO2___

PO2___

Bicarbonate___

Antibiotics given in the ICU________

1. Is the patient on ventilator support: Yes or No

**Key:**

PCO2-arterial carbon dioxide partial pressure

PO2-Arterial oxygen partial pressure

bpm-beats per minute

brpm-breath per minute

IHK international hospital Kampala
